# Supplementary material for: Updating estimates of Plasmodium knowlesi malaria risk in response to changing land use patterns across Southeast Asia
Source: PLoS Negl Trop Dis. 2024 Jan 22;18(1):e0011570. doi: 10.1371/journal.pntd.0011570 (PMC10833542; doi:10.1371/journal.pntd.0011570)
Supplement: S1 Text — Figure A in S1 Text. Summary statistics for modelled transmission suitability across Southeast Asia, calculated across the set of 500 bootstraps. Results are only displayed where an area is in the range of both a vector and reservoir species necessary for transmission (see Methods). Administrative boundary base maps sourced from the Malaria Atlas Project (CC BY 3.0, [32]) and international boundaries from the US Department of State Large Scale International Boundaries dataset (public domain, [33]). Figure B in S1 Text. Predicted mean transmission suitability with overlay of infection occurrence data across the evaluation region. Results are only displayed where an area is in the range of both a vector and reservoir species necessary for transmission (see Methods). A: Predicted transmission suitability with infection occurrence polygons and points in blue. B: Predicted transmission suitability with infection absence polygons in blue. Administrative boundary base maps sourced from the Malaria Atlas Project (CC BY 3.0, [32]) and international boundaries from the US Department of State Large Scale International Boundaries dataset (public domain, [33]). Figure C in S1 Text. Comparison of the modelled mean transmission suitability value between the current work and the 2015 model. Model predictions are presented from the current work with data as of 2020 (A), and the predictions presented in the 2015 model [31] (B). Note that the absolute value of predictions are not necessarily comparable given differences in model specification and training data. Results are only displayed where an area is in the range of both a vector and reservoir species necessary for transmission (see Methods). Administrative boundary base maps sourced from the Malaria Atlas Project (CC BY 3.0, [32]) and international boundaries from the US Department of State Large Scale International Boundaries dataset (public domain, [33]). Figure D in S1 Text. Changes in the distribution of predicted transmissio [file pntd.0011570.s001.pdf]

Supplementary Materials — Updating estimates of *Plasmodium knowlesi* malaria risk in response to changing land use patterns across Southeast Asia

Ruarai J Tobin[1, †], Lucinda E Harrison[2], Meg K Tully[1], Inke N D Lubis[3], Rintis Noviyanti[4], Nicholas M Anstey[5], Giri S Rajahram[6], Matthew J Grigg[5], Jennifer A Flegg[2], David J Price[1,7], and Freya M Shearer[1,8, †]

[1]Infectious Disease Dynamics Unit, Melbourne School of Population and Global Health, The University of Melbourne, Melbourne, Australia
[2]School of Mathematics and Statistics, The University of Melbourne, Melbourne, Australia
[3]Department of Paediatrics, Faculty of Medicine, Universitas Sumatera Utara, Medan, Indonesia
[4]Eijkman Research Center for Molecular Biology, BRIN, Jakarta, Indonesia
[5]Menzies School of Health Research and Charles Darwin University, Darwin, Australia
[6]Infectious Diseases Society Kota Kinabalu Sabah, Menzies School of Health Research Clinical Research Unit, Hospital Queen Elizabeth II, and Clinical Research Centre, Queen Elizabeth Hospital, Ministry of Health, Kota Kinabalu, Malaysia
[7]Doherty Institute for Infection and Immunity, The Royal Melbourne Hospital and The University of Melbourne, Melbourne, Australia
[8]Infectious Disease Ecology Modelling Group, Telethon Kids Institute, Perth, Australia
[†]Corresponding authors: Ruarai J Tobin (ruarai.tobin@unimelb.edu.au) and Freya M Shearer (freya.shearer@unimelb.edu.au)

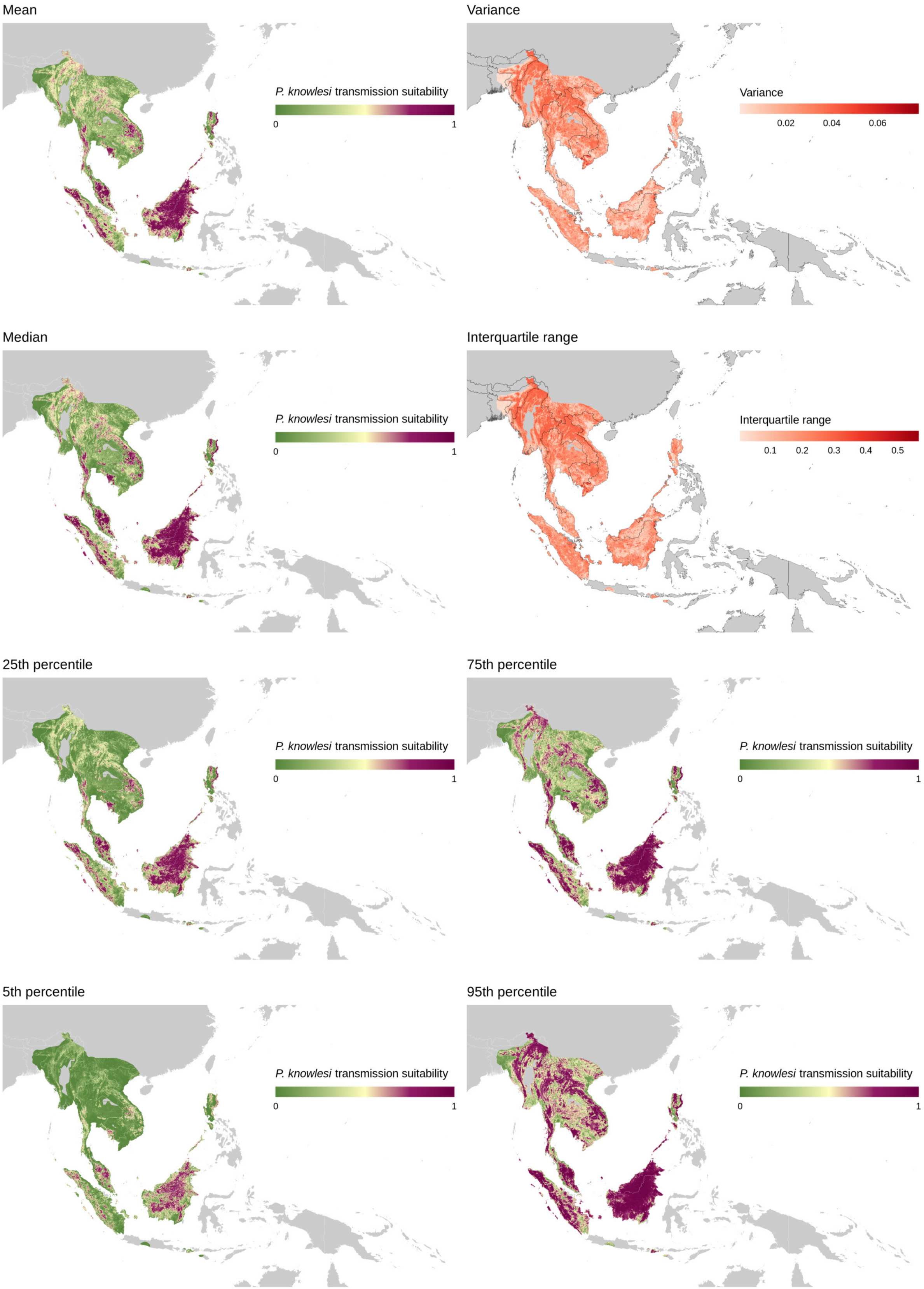

Figure A: **Summary statistics for modelled transmission suitability across Southeast Asia, calculated across the set of 500 bootstraps.** Results are only displayed where an area is in the range of both a vector and reservoir species necessary for transmission (see Methods).

Administrative boundary base maps sourced from the Malaria Atlas Project (CC BY 3.0, [1]) and international boundaries from the US Department of State Large Scale International Boundaries dataset (public domain, [2]).

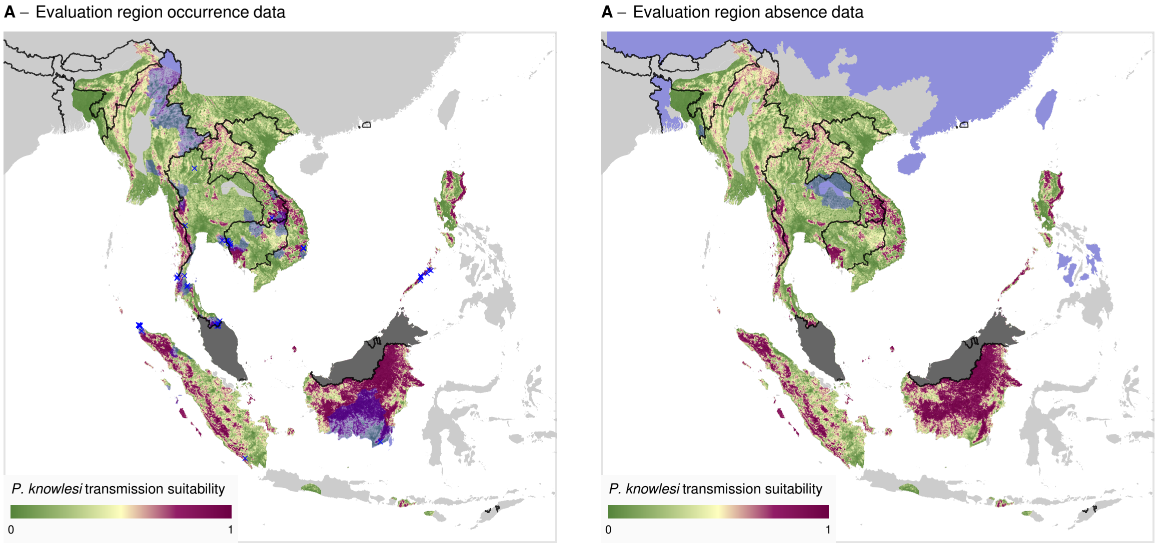

Figure B: **Comparison of the modelled mean transmission suitability value between the current work and the 2015 model.** Model predictions are presented from the current work with data as of 2020 (**A**), and the predictions presented in the 2015 model [3] (**B**). Note that the absolute value of predictions are not necessarily comparable given differences in model specification and training data. Results are only displayed where an area is in the range of both a vector and reservoir species necessary for transmission (see Methods).

Administrative boundary base maps sourced from the Malaria Atlas Project (CC BY 3.0, [1]) and international boundaries from the US Department of State Large Scale International Boundaries dataset (public domain, [2]).

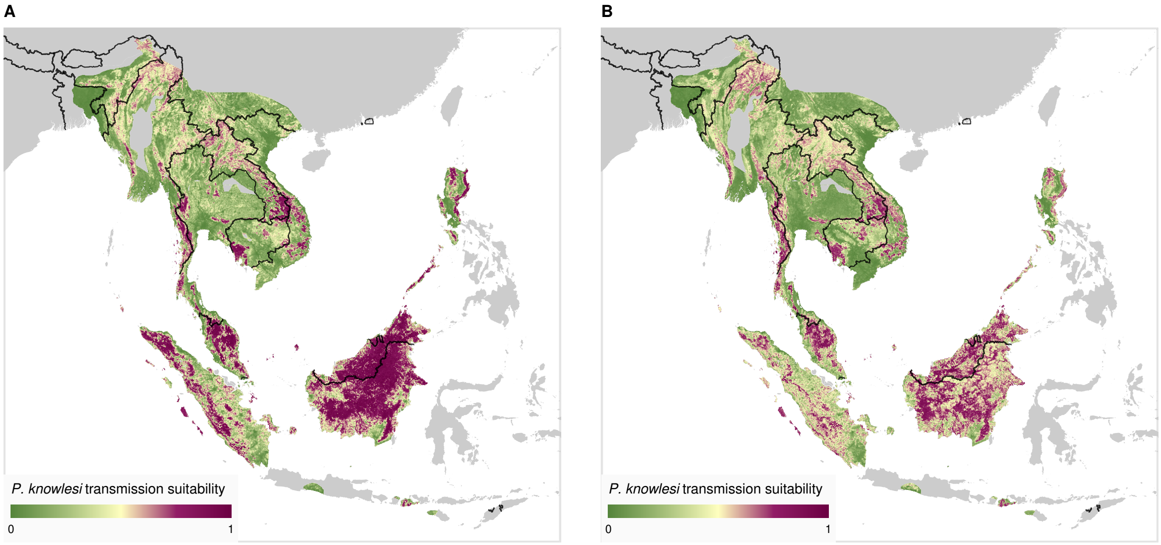

Figure C: **Comparison of the modelled mean transmission suitability value between the current work and the 2015 model.** Model predictions are presented from the current work with data as of 2020 (**A**), and the predictions presented in the 2015 model [3] (**B**). Note that the absolute value of predictions are not necessarily comparable given differences in model specification and training data. Results are only displayed where an area is in the range of both a vector and reservoir species necessary for transmission (see Methods).

Administrative boundary base maps sourced from the Malaria Atlas Project (CC BY 3.0, [1]) and international boundaries from the US Department of State Large Scale International Boundaries dataset (public domain, [2]).

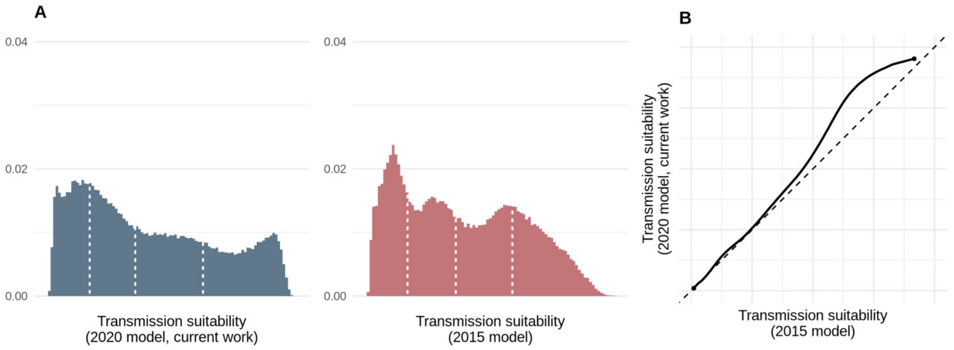

Figure D: **Changes in the distribution of predicted transmission suitability between the current study and the 2015 model.** Histograms (**A**) and quantile-quantile plot (**B**) comparing the distributions of mean predicted transmission suitability for the 2015 and 2020 models of *P. knowlesi* transmission risk. Histograms are presented on a relative x-axis (ranging from minimal to maximal predicted mean risk), with quartiles of predicted risk displayed as dashed vertical lines.

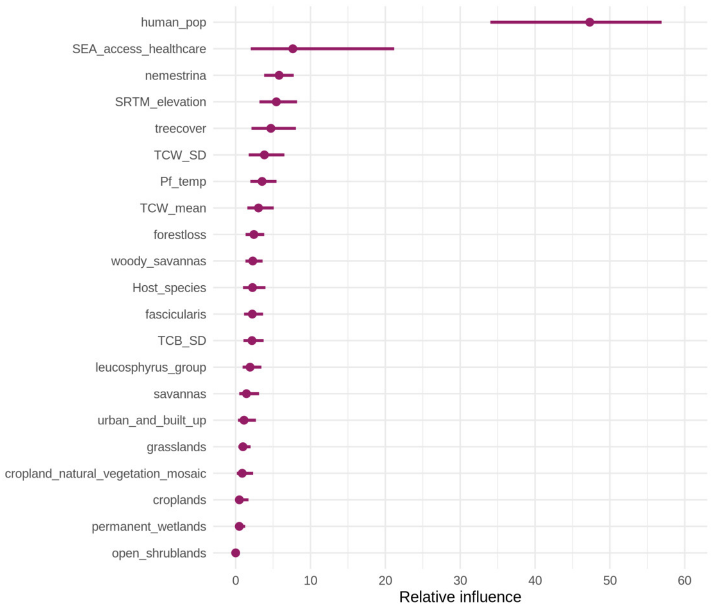

Figure E: **Relative influence scores for each covariate.** Scores are calculated for each bootstrap, with points and lines representing median values and 95% confidence intervals respectively.

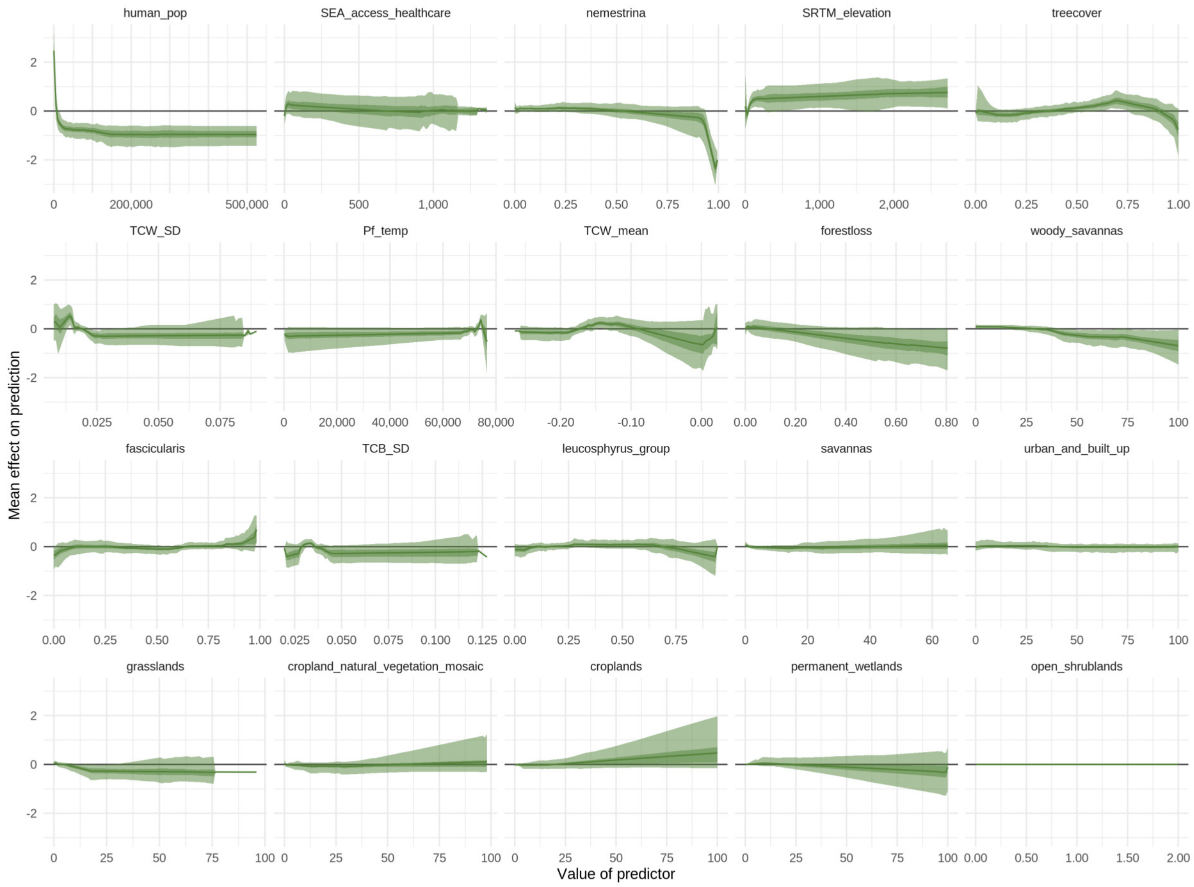

Figure F: **Accumulated local effect (ALE) plots for each covariate.** ALE indicates the mean effect of changing a covariate's value upon the prediction (on logistic scale) across the range of that covariate. The ALE values are calculated for each bootstrap, with the median value, 50% and 95% confidence intervals presented as lines, darker shaded regions and lighter shaded regions respectively.

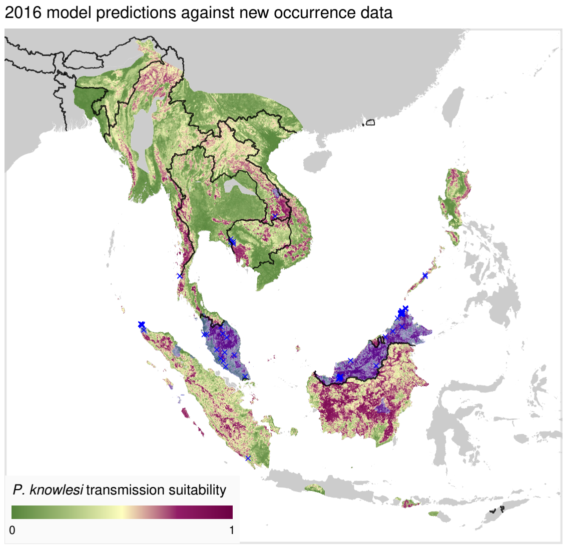

Figure G: **Comparison of predicted transmission risk as presented in the 2016 work and the occurrence data collected in the 2020 literature review.** Results are only displayed where an area is in the range of both a vector and reservoir species necessary for transmission (see Methods).

Administrative boundary base maps sourced from the Malaria Atlas Project (CC BY 3.0, [1]) and international boundaries from the US Department of State Large Scale International Boundaries dataset (public domain, [2]).

	Human	Macaque	Mosquito	Totals	
Country/Region	n	n	n	2020	(2015)
Brunei	0	0	0	0	(6)
Cambodia	5	0	0	5	(6)
Indonesia (Total)	44	1	0	45	(5)
Kalimantan Selatan	0	0	0	0	(3)
Kalimantan Tengah	1	0	0	1	(2)
Lampung	0	1	0	1	(0)
Nanggroe Aceh Darusalam	36	0	0	36	(0)
Sumatera Utara	7	0	0	7	(0)
Laos	3	0	0	3	(1)
Malaysia (Total)	181	8	12	201	(183)
Johor	3	0	0	3	(3)
Kedeh	2	0	0	2	(1)
Kelantan	5	0	0	5	(17)
Melaka	2	0	0	2	(1)
Negeri	2	0	2	4	(3)
Pahang	3	0	1	4	(11)
Perak	3	0	1	4	(1)
Pulau Pinang	1	0	0	1	(1)
Sabah	127	7	0	134	(60)
Sarawak	22	1	5	28	(52)
Selangor	7	0	3	10	(30)
Terengganu	1	0	0	1	(2)
W.P. Kuala Lumpur	3	0	0	3	(0)
W.P. Labuan	0	0	0	0	(1)
Myanmar	2	0	0	2	(3)
Philippines	1	0	2	3	(7)
Singapore	0	0	0	0	(6)
Thailand	4	0	0	4	(32)
Vietnam	1	0	0	1	(11)

Table A: **The number of human, macaque and mosquito samples in the occurrence database produced by the 2015 literature review.** Samples in Indonesia and Malaysia are shown stratified by region (province, state or territory). Total counts are shown for records from both the 2020 literature review and 2015 literature review.

References

[1] Daniel A Pfeffer, Timothy C D Lucas, Daniel May, Joseph Harris, Jennifer Rozier, Katherine A Twohig, Ursula Dalrymple, Carlos A Guerra, Catherine L Moyes, Mike Thorn, Michele Nguyen, Samir Bhatt, Ewan Cameron, Daniel J Weiss, Rosalind E Howes, Katherine E Battle, Harry S Gibson, and Peter W Gething. malariaAtlas: an R interface to global malariometric data hosted by the Malaria Atlas Project. *Malar. J.*, 17(1):352, October 2018.

[2] The Office of the Geographer and Global Issues at the U.S. Department of State. Large Scale International Boundaries (LSIB). `https://data.humdata.org/dataset/large-scale-international-boundaries-lsib`. Accessed: 2022-4-14.

[3] Freya M Shearer, Zhi Huang, Daniel J Weiss, Antoinette Wiebe, Harry S Gibson, Katherine E Battle, David M Pigott, Oliver J Brady, Chaturong Putaporntip, Somchai Jongwutiwes, et al. Estimating geographical variation in the risk of zoonotic *Plasmodium knowlesi* infection in countries eliminating malaria. *PLOS Neglected Tropical Diseases*, 10(8):e0004915, 2016.
