# Supplementary material for: Updating estimates of Plasmodium knowlesi malaria risk in response to changing land use patterns across Southeast Asia
Source: PLoS Negl Trop Dis. 2024 Jan 22;18(1):e0011570. doi: 10.1371/journal.pntd.0011570 (PMC10833542; doi:10.1371/journal.pntd.0011570)
Supplement: Attachment — Submitted filename: response_19Dec2023.pdf [file pntd.0011570.s002.pdf]

Dear PLOS Neglected Tropical Diseases Editorial Board,

Many thanks for your email on November 14, 2023 and your invitation to revise and submit our article (PNTD-D-23-00992) for further consideration by PLOS Neglected Tropical Diseases.

We found the comments and suggestions from the reviewers to be very helpful and have revised the manuscript. In line with these comments, our revision includes clarifications regarding our methodology, the presentation of results and the potential impact of spatially biased underascertainment. We have also extended the discussion of our modelling work as it relates to the previously published 2015 model of *P. knowlesi* transmission risk.

Please find below our point-by-point response to the reviewer comments.

Note that we have also made an adjustment to our study prediction area. We previously allowed our model to predict transmission suitability across the Indonesian island of Sulawesi (as per the 2015 analysis) given the potential for transmission of *P. knowlesi* within a reservoir of pet macaques. Following further consultation with research consortium colleagues and public health stakeholders in Indonesia (which occurred after the manuscript submission), we have now excluded Sulawesi from our prediction area. We believe that this is the most accurate representation of risk across the region. Please find the attached figure below which displays the change in prediction extent.

We thank the reviewers and editors again for their input and consideration of this article, and feel that the manuscript is improved as a result. We would be happy to answer any further questions that you might have and look forward to hearing your decision.

With thanks,
Mr Ruarai J Tobin and Dr Freya M Shearer,
The University of Melbourne

On behalf of all authors

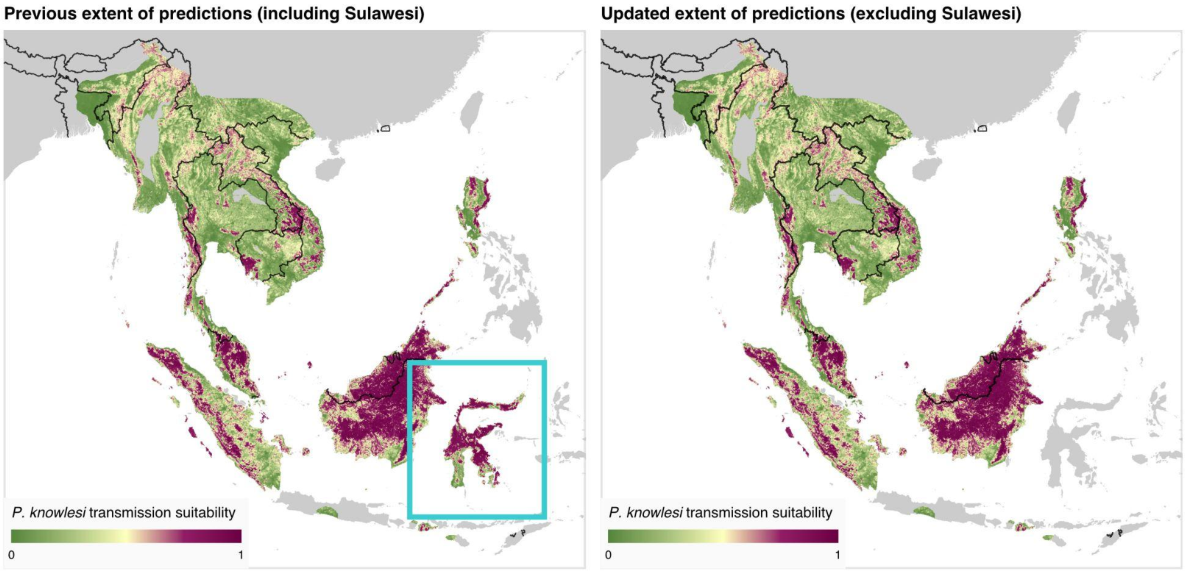

Updated extent of *P. knowlesi* transmission suitability predictions across Southeast Asia. The region of Sulawesi, Indonesia has been excluded from our predictions in response to additional post-submission consultation with study consortium colleagues and public health stakeholders.

Methods

Reviewer #1: The methods outlined are reasonable and sufficient for the analysis undertaken. The conform to a number of standards in the field. I have no issues with the methodology in the form presented, and while there are possible extensions, I have found that they make little difference to the results, and they are not necessary to undertake in all instances.

We thank the reviewer for their thorough examination of the methodology.

Reviewer #2: I agree BRTs are an appropriate tool for this sort of modelling. But given this work is set into context of increasing risk, I think some discussion about the static nature of the presented results [Fig3A] is necessary: is the presented transmission suitability a mean over the time period of data collection, or contemporary suitability?

To clarify the timing of the *Plasmodium knowlesi* transmission suitability predictions presented, we have amended the methods (line 233):

> Predictions were made using covariate data corresponding to 2019, the most recent year available.

and results (line 297):

> Predictions were produced using covariate raster datasets as of 2019, representing our most up-to-date estimate of transmission suitability across the region.

Background points [L205]: I think this needs clarification – is the assumption that more populated areas are more likely to report cases for a given incidence, or that incidence is higher and therefore cases are more likely to occur? I would imagine more urban populations would be at lower risk of Knowlesi infection.

We agree with the reviewer that urban populations would be expected to be at lower risk of *Plasmodium knowlesi* infection. In our model, we sampled background points proportional to human population density in order to adjust for the potential bias of a higher case detection rate in urban areas, i.e. the first assumption that the reviewer has described. We have modified the section (line 198) to clarify this point:

> ~~To produce background points for the human and mosquito records, as in the 2015 model [31], we sampled points across the training region, with this sampling weighted by human population density [41] under the assumption that more populous areas would have a greater probability of reporting human cases and that the locations of mosquito infection studies were selected based on the presence of human *P. knowlesi* cases.~~

> Background points were produced as in the 2015 analysis [31]. To produce the human record background points, we sampled points across the training region weighted by human population density [41], under the assumption that human *P. knowlesi* infections would be more likely to be detected and reported within more populous areas. Background points for mosquito records were similarly produced with sampling weighted by human population density, under the assumption that the locations of mosquito infection studies would be selected based on the presence of human *P. knowlesi* cases.

Similarly, for the macaque background records [L210/11] – assuming sub-microscopic disease to be uniform geographically needs a citation, ideally.

We have removed the claim made on line 210/11 and instead review the possibility of non-uniform spatial distribution of asymptomatic or sub-microscopic disease and the potential effects on our results in the discussion on line 475:

> In our approach, we implicitly assume that under-ascertainment of *P. knowlesi* infections in humans due to asymptomatic/submicroscopic or spontaneously resolving disease (see [9, 10, 11]) has a uniform effect geographically. If the data used for this study were biased by such under-ascertainment which was not uniform across space (e.g. due to differing levels of immunity between regions), this would be expected to in turn bias our predicted transmission suitability downwards in environments similar to those where under-ascertainment were occurring. Further research on any potential spatial association of asymptomatic or submicroscopic human *P. knowlesi* infection would be of high value in further refining estimates of the spatial distribution of transmission of the parasite.

[L218-220] - what does 'degraded' mean in this context? Generally, I don't think this explanation is very clear to a more general audience, and Figure 2A doesn't particularly help. It is not clear to me how polygon and pixel data have been combined in this framework.

We have updated the text to clarify how we produce occurrence points from occurrence records across each bootstrap:

> ~~We then degraded the occurrence polygon records sampled to points via spatially uniform sampling of a singular point across the set of points bounded by each polygon (Figure 2A)~~
>
> Each polygon occurrence record was then reduced to a single point location; this was achieved by selecting a point at random uniformly across each polygon for each bootstrap. As areas of overlapping polygons therefore have a greater probability of a point being sampled, we present the density of overlapping polygons in Figure 2A.

Results

Reviewer #1: Given that this is an updating of a prior analysis, I was very surprised to see how little the prior analysis factored into the results. Why was the update required now? Why not a year prior, why not a year later? How good were the old predictions in the context of the new occurrence data? Was incorrect prediction a trigger for re-running? If the objective is for stakeholders to use these maps to make decisions with, demonstrating that prior versions were or were not sufficient to make similar decisions is a critically important perspective to provide. Should I wait until 2030 for the next iteration before I am finally confident that things are stable? We have to make decisions today - how confident can I be in making those decisions with this resource, given the past performance of the prior analysis?

These are important points and we agree that further discussion of these would improve the manuscript. In regards to the performance of the predicted *P. knowlesi* transmission suitability presented in the 2015 work, we have added a new figure comparing these predictions against the newly collected occurrence data (Figure S7). We have included the following text on line 327 in the results referring to this qualitative performance analysis:

> We additionally examine the performance of the predicted *P. knowlesi* transmission risk map presented by the 2015 analysis against the occurrence data collected in our literature review (Figure S7). We find that, qualitatively speaking, the performance of the 2015 analysis in predicting the presence of infection occurrences published in the literature between October 2015 and March 2020 was good.

The decision to produce an updated model of *P. knowlesi* transmission risk was prompted by the accumulation of newly collected infection occurrence data, with the expectation that such data would improve model estimates, and the recognition that ongoing land cover change due to deforestation may have induced changes in the underlying spatial distribution of risk. Assessing the sufficiency of model predictions in a context of a potentially changing distribution of risk and ongoing data collection is not straightforward. Ideally, the decision to produce updated geospatial risk models would be guided by quantitative evidence.

An analysis of the sufficiency of our predictions may be particularly difficult due to the nature of *P. knowlesi* infection occurrence data. Such occurrence data has historically been collected sporadically, with a mixture of prospective sampling efforts and localised surveillance programs comprising the majority of the data used in our study. These sampling efforts occur within a context of a potentially changing underlying spatial distribution of infection risk. Disentangling these factors would likely be a highly complex task and is beyond the scope of our study. If systematic sampling efforts were to be performed (e.g. as incorporated in modelling by the Malaria Atlas Project), the potential for change in the underlying distribution of risk could be isolated and the sufficiency of a static prediction evaluated.

We now provide discussion of these points at line 397:

> Updating a model of risk — as we have performed here for *P. knowlesi* transmission suitability — raises key questions regarding when and why such an update should be performed. We produced the update in response to two primary factors: the accumulation of further *P. knowlesi* infection occurrence data since the publication of the previous mapping study, which was expected to improve estimate precision when incorporated into the model; and changes in land cover across Southeast Asia, such as deforestation, which were suspected to have caused changes in the underlying distribution of transmission risk. For our predictions (with covariate data as of 2019 and occurrence data up to 2018) to be sufficient for future sampling efforts, the change in the underlying distribution of risk over time should be minimal. However, identifying if such changes have occurred is particularly difficult for *P. knowlesi* given the nature of data collection for the pathogen. Whereas studies of the human malaria species are able to isolate the effect of a changing risk distribution through the use of data which has been collected in a systematic manner [72], the data available for our study largely comprises infection occurrences identified by localised prospective sampling and passive surveillance. Despite these difficulties, we note that the good predictive performance of the 2015 analysis (Figure S7) provides reason to believe that our static estimates are sufficient to inform future sampling efforts.

The guidance provided in the 2015 analysis largely focussed on the need for further surveillance in Myanmar, Laos, Sumatra, Kalimantan and Palawan. We note although newly collected infection occurrence data was present within each of these regions, all but Palawan still appear broadly unsampled. We have expanded on the comparison of our results to the 2015 predictions on line 366:

> In contrast, Palawan in The Philippines was also highlighted as a target for future sampling efforts in the 2015 analysis [31], with new occurrence records confirming the presence of *P. knowlesi* in this region [60].

Reviewer #2: The results are coherently presented.

We thank the reviewer for their considered feedback.

Transmission suitability [L280]. Please define this metric when it's introduced, as it means different things to different audiences and it's not clear quite what is meant until line 304 down the page.

We agree with the reviewer that our use of this term here requires further clarification. We have updated the initial reference to transmission suitability to include a short definition of the metric:

> ~~The mean and standard deviation of predicted *P. knowlesi* transmission suitability across at-risk areas of Southeast Asia is presented in Figure 3.~~
>
> The mean and standard deviation of predicted *P. knowlesi* transmission suitability (a relative measure of the potential risk of *P. knowlesi* transmission to humans) across at-risk areas of Southeast Asia is presented in Figure 3.

Conclusions

Reviewer #1: I don't think the main conclusions are too different from the prior modelling exercise, in spite of the new data and covariates. I do not think this is a bad thing - instead this provides a very unique opportunity to retrospectively evaluate the value of the prior exercise in the context of the new data, and justify whether the new methods changes were necessary. Allowing readers to appreciate when, or not, a model is worth re-doing, and how to track that ongoing performance is key. Areas in northern Myanmar are very different in the environmental suitability index score [although direct comparison of this index value with 2015 index values is to be strongly cautioned]. Are any of the guidance from the 2015 analysis found to be different in the context of the data and methods upgrades? When should the next assessment be done? Does it require data (if so, in what places?), does it require covariates to change (e.g. climate change shifting things in a way not observed previously?), do we just wait 8 years? What should we be looking out for as concerning enough to prompt a new model run?

The reviewer's comments regarding the primary conclusions are highly appreciated. We agree that including further discussion of when and if an updated model prediction should be produced is of significant value. We have combined our response to these comments with the response above (Results, Reviewer 1).

Reviewer #2: The conclusions of the paper are supported by the results, and the limitations are clearly described.

We thank the reviewer for their considered feedback.

Discussion [L378-387]: Are you able to explain why (even speculatively) these changes have occurred since the 2015 work? EG do the covariates look substantially different?

Unfortunately, within the correlative modelling framework used for this work, determining the causes of differences in model outputs is particularly difficult. We attempted to identify such potential causes

throughout the course of the study, however, this did not yield clear results. We have amended the discussion to include a reflection on these difficulties at line 453:

> Identifying potential reasons for such changes in model output is also difficult. Such an analysis would require a systematic examination of differences in model structure, covariate data and occurrence data, and, given the correlative nature of the model, would not necessarily be expected to provide insight into the mechanisms of *P. knowlesi* risk.

Editorial and Data Presentation Modifications

Reviewer #1: (No Response)

Reviewer #2: Minor comment: Why a diverging (rather than sequential) colour ramp for Figure 3A?

Although a sequential colour ramp would also be suitable for this figure, we believe that a diverging colour ramp allows for easier identification of the regions of both high or low predicted suitability (in green and magenta) and intermediate predicted suitability (in yellow). Additionally, this colour ramp means that the presentation of our results is consistent with that in the original 2015 analysis.
